# Simulation and optimization of 30.17% high performance N-type TCO-free inverted perovskite solar cell using inorganic transport materials

**DOI:** 10.1038/s41598-024-62882-7

**Published:** 2024-05-26

**Authors:** Emmanuel A. Nyiekaa, Timothy A. Aika, Eli Danladi, Christopher E. Akhabue, Patience E. Orukpe

**Affiliations:** 1 Department of Electrical and Electronics Engineering, University of Benin, Benin City, Nigeria; 2 Department of Physics, Federal University of Health Sciences, Otukpo, Nigeria; 3 Department of Chemical Engineering, University of Benin, Benin City, Nigeria; 4 Department of Electrical and Electronics Engineering, Joseph Sarwuan Tarka University, Makurdi, Nigeria

**Keywords:** SCAPS-1D software, Perovskite solar cell, Hole transport layer, Light absorber, Electron transport layer, Materials science, Nanoscience and technology

## Abstract

Perovskite solar cells (PSCs) have gained much attention in recent years because of their improved energy conversion efficiency, simple fabrication process, low processing temperature, flexibility, light weight, and low cost of constituent materials when compared with their counterpart silicon based solar cells. Besides, stability and toxicity of PSCs and low power conversion efficiency have been an obstacle towards commercialization of PSCs which has attracted intense research attention. In this research paper, a Glass/Cu_2_O/CH_3_NH_3_SnI_3_/ZnO/Al inverted device structure which is made of cheap inorganic materials, n-type transparent conducting oxide (TCO)-free, stable, photoexcited toxic-free perovskite have been carefully designed, simulated and optimized using a one-dimensional solar cell capacitance simulator (SCAPS-1D) software. The effects of layers’ thickness, perovskite’s doping concentration and back contact electrodes have been investigated, and the optimized structure produced an open circuit voltage (V_oc_) of 1.0867 V, short circuit current density (J_SC_) of 33.4942 mA/cm^2^, fill factor (FF) of 82.88% and power conversion efficiency (PCE) of 30.17%. This paper presents a model that is first of its kind where the highest PCE performance and eco-friendly n-type TCO-free inverted CH_3_NH_3_SnI_3_ based perovskite solar cell is achieved using all-inorganic transport materials.

## Introduction

Due to the enormous energy demand brought on by the growing world population, energy consumption is increasing at fast rate every day. Fossil fuels provide about 80% of the world’s energy requirements^1^, which have negative environmental effects and have forced the development of renewable energy sources^2^. Because of its abundant, clean, and limitless nature, solar energy is regarded as the most major and significant source of renewable energy^3^, making it the most promising contender because of its affordability and sustainability^4^. The generations of solar cells is recently categorized into four; the crystalline silicon (c-Si) and gallium arsenide (GaAs) constitute the first generation, while the thin films such as copper indium gallium selenide (CIGS), amorphous silicon (a-Si) and cadmium telluride (CdTe), formed the second generation. The evolving class of solar cells such as dye-sensitized solar cells (DSSC), copper zinc tin sulfide (CZTS) and quantum dot (QD) belongs to the third generation while the novel solar cells recognized as “inorganics-in-organics” such as hybrid perovskites represents one of the candidates of fourth-generation solar cells^5^. The monocrystalline and polycrystalline silicon-based are the known two kinds of solar cells^6^. Solar cells based on CdTe^7,8^, quantum dot sensitized-based solar cells^9^, CIGS^10,11^, organic photo cells^12^ and perovskite-based solar cells^13^ have also been explored by researchers.

The metal halide perovskites are represented by ABX_3_, where A refers to an organic cation, B refers to a metal cation, and X refers to a halogen anion. The cation embraces individual or mixed compositions of methylammonium (MA), cesium (Cs), and formamidinium (FA), whereas the halogen anion embraces individual or mixed compositions of Cl, Br, and I^14^. Recent researches have focused on perovskite solar cells (PSCs), due to their increased efficiencies^15^, low processing temperatures, high absorption, long diffusion length, high charge mobility, low trap density, low exciton binding energy, tunable bandgap and low-cost of fabrication^14,15^. PSCs have been the subject of numerous studies, which have improved energy power conversion efficiencies (PCEs) from 3.8% in 2009 to about 25% after 13 years of development^16^, while^15^ reported PCE of 25.6%, and 26.1% have recently been attained^17^. The impressive characteristics of perovskite materials include good charge carrier mobility, high coefficient of absorption, high diffusion charge carrier and low binding energy^18,19^. Because of their excellent photovoltaic performance, methyl ammonium lead halides, both pure and modified, have been the subject of several studies^20,21^. However, lead-based electronics posed hindrance to commercialization due to its harmful nature^14,22–24^. Recent reports revealed stern circumscribe of lead-based electronics devices by many countries, notably the European Union because of its toxic nature to human and environment irrespective of their high-power conversion efficiency^25–28^.

Some of the major challenges affecting the large-scale production of PSCs is the high cost of electron and hole transport materials, toxicity of the perovskite materials and degradability of the solar cells. Despite significant improvements made thus far, high temperature and humidity as well as the presence of moisture results to reduced lifespan of some perovskite materials such as Spiro-OMeTAD, the popular hole transport material. The difficulty in processing, and the expensive nature of Spiro-OMeTAD is a possible impediment to commercialization of PSCs going forward^29–32^. Additionally, the Spiro-OMeTAD layer aids polarisation of the electrode and plays a significant part in the current density–voltage (J–V) hysteresis phenomenon, which ultimately influence the device’s instability^33^. Conventional structures of PSCs using organic-based materials as hole transport medium and the metallic electrodes constitute the major reasons for the PSCs’ shorter life span^34^. Pin-holes in the HTM have been a challenge of interest in recent studies as it leads to poor PSC’s stability owing to penetration of oxygen and ambient moisture which deteriorates the perovskite absorbent layer. Numerous attempts have been made to address the detrimental effects of pinholes in HTMs and perovskites. One of such efforts is the significant improvement of stability in perovskite solar cells through the use of doping engineering to create a hole transport layer free of pinholes^35^. Investigations by other scientists have concentrated on creating effective PSCs employing novel kinds of hole-transport materials as replacement to Spiro-OMeTAD^36,37^, or PSCs without HTL that are suitable for streamlining the device’s ideal process, and further reduce manufacturing cost and as well prevents perovskite’s degradation^38–40^. There is no doubt that the absence of pinholes in HTM layer considerably increases the PSC’s device stability under operating environments^41^.

Regardless of tremendous research progresses in PCSs, planar inverted PSCs have received lesser interest, hence limited research work is conducted in this area of study despites their easy fabrication, cost-effectiveness, and suppressed hysteresis characteristics^42^. Therefore, intense study is required in this field of study to improve and maximize their performances as compared to their conventional counterparts’ structures. Most of the available researches on inverted planar PSCs have focused on the use of gold as contact electrode, spiro-OMeTAD as HTM with a maximum achievable PCE of approximately 30% through simulations^43–47^.

This research sought to streamline production innovations in PSCs, lower production costs, and maximise performance. The use of eco-friendly perovskite material, cheap and suitable all-inorganic transport materials and the use of the appropriate back contact electrode will increase efficiency, stability, and significantly reduce the production costs. The possibility of attaining high efficiency by utilising the inverted planar technique without using silicon composites and organic HTMs can lead to a greater variety of benefits in the sector. This paper presents a simulation of an inverted planar and n-type transparent conducting oxide-free structure using inorganic transport materials. The selection of appropriate back contact electrode, variation of system’s parameters such as thickness of HTM, absorber material, ETM, absorber doping concentration and determination of optimal values of series and shunt resistances is carried out to achieve optimal performance of the device.

## Materials and methods

### Device structure and simulation

There are different types of software used for simulation of solar cells such as PC1D, ASA, Amps-1D, WxAMPS, SCAPS-1D, SETFOS, Gpvdm, AFORS-het, Aspin-2D, PECSIM, Adept, TCAD, Atlas, Silvaco etc. However, SCAPS-1D software is used in this work to simulate an inverted tin-based perovskite solar cell with planar heterojunction because of its best accurate non-commercial tool that is straightforward in operation, with friendly dialog box and extremely quick in simulations at no additional expense and support for multi-junction solar cells^48^. Three related differential equations were solved to determine the energy bands, quantum efficiency of the device, current density–voltage (J–V) curve, and recombination rate curve. The Poisson Eq. (1), the electron continuity Eq. (2), and the hole Eq. (3) are built in the SCAPS-1D software. These curves are used to compute the solar cell device’s open circuit voltage (V_oc_), short circuit current density (J_SC_), fill factor (FF), and power conversion efficiency (PCE).

1 $$\frac{d}{dx}\left(-\varepsilon(x)\frac{d\psi}{dx}\right)=q\left[p\left(x\right)-n\left(x\right)+{N}_{D}^{+}\left(x\right)-{N}_{A}^{-}+{p}_{t}\left(x\right)-{n}_{t}\left(x\right)\right]$$

2 $$\frac{{dn}_{p}}{dt}={G}_{n}-\frac{{n}_{p}-{n}_{p0}}{{\tau }_{n}}+{n}_{p}{\mu }_{n}\frac{dE}{dx}+{\mu }_{n}E\frac{{dn}_{p}}{dx}+{D}_{n}\frac{{d}^{2}{dn}_{p}}{{dx}^{2}}$$

3 $$\frac{{dp}_{n}}{dt}={G}_{p}-\frac{{p}_{n}-{p}_{n0}}{{\tau }_{p}}+{p}_{n}{\mu }_{p}\frac{dE}{dx}+{\mu }_{p}E\frac{{dp}_{n}}{dx}+{D}_{p}\frac{{d}^{2}{dp}_{n}}{{dx}^{2}}$$

where $G$, ${\tau }_{n}$, ${\tau }_{p},$ $D$, $q$, $\varepsilon$  $\psi,$ ${\mu }_{n}$, ${\mu }_{p}$, $n\left(x\right), p\left(x\right),$ ${n}_{t}(x)$, ${p}_{t}\left(x\right),$ ${N}_{A}^{-}(x),$ ${N}_{D}^{+}\left(x\right)$ and $E$ represent the rate of generation, life time of electron, life time of hole, diffusion coefficient, electron charge, permittivity, electrostatic potential, electron mobility, hole mobility, concentration of free electrons, concentration of free holes, concentration of trapped electrons, concentration of trapped holes, ionized acceptor concentrations, ionized donor concentrations, and electric field respectively. Meanwhile, x represents the direction along the thickness of the solar cell^49^.

The device’s structure is composed of Cu_2_O as HTM layer, CH_3_NH_3_SnI_3_ as absorber layer and ZnO as ETM layer. The choice of Cu_2_O as HTM in this work over other HTMs is based on the fact that it is relatively cheap when compared to organic based HTMs, high absorption coefficient, high intrinsic hole mobility, and acceptable energy levels that are aligned with the absorber layer (MASnI_3_), high photochemical and thermal stability as well as long-term stability in air^50^. Similarly, CH_3_NH_3_SnI_3_ is also adopted as the absorber layer because of its eco-friendliness with potentials for commercialization^51,52^ and superior optoelectronic properties with 1.3 eV direct band gap, which is an appropriate range for the absorber layer^21,49,53,54^. Meanwhile, ZnO is used as ETM because of its high absorption coefficient, higher electron mobility^2,51^ and aligned energy bandgap with the chosen perovskite (MASnI_3_) when compared to the SnO_2_^36^.

### Background and selection of device parameters

Light generates electron–hole pairs within the absorber layer. The junction field draws holes to the HTM layers and electrons to the ETM layers, respectively. The thickness, coefficient of absorption, and mobility of the active material all affect the device’s J_SC_. The photocurrent will increase as the absorption coefficient increases^49,54,55^. Another important consideration is the absorber’s thickness, which must be sufficient to absorb the maximum cutoff wavelength of the incident solar light^49,53^. Aside from that, mobility is essential to achieving the high J_SC_ which is ideally equal to the current in the solar cell. For the sample of CH_3_NH_3_SnI_3_ produced using the open tube approach, a very high mobility of electrons (2000 cm^2^/Vs) and holes (300 cm^2^/Vs) was discovered by Ma et al. and Stoumpos et al.^56,57^. Lazemi et al. reported a high value of J_SC_ using similar values of carrier mobility^53^. Devi et al.^58^ and Khattak et al.^59^ have taken into account the equal and noticeably lesser values of the electron and hole mobility, which are 1.6 cm^2^/Vs and 0.16 cm^2^/Vs respectively. In line with experimental work done by^60^, the electron (2000 cm^2^/Vs) and hole (300 cm^2^/Vs) mobility values for CH_3_NH_3_SnI_3_ is adopted for use in this study. It is important to note that diffusion length also has a proportionality relationship to the square root of mobility^58^.

The device simulation was conducted under the 1000 W/m^2^ light illumination at 300 K temperature and 1.5G air mass. The proposed device’s series resistance was adjusted to 1 Ωcm^2^ while the shunt resistance at 10^4^ Ωcm^2^ during simulation. The value of work function for front electrode (Cu_2_O) is 5.0 eV while the surface recombination velocity for electrons and holes as 10^5^ cm/s and 10^7^ cm/s respectively. Moreover, the work function for the back contact electrode ticked as flat band with surface recombination velocity for electrons and holes as 10^7^ cm/s and 10^5^ cm/s respectively at the beginning of the simulation until an optimized back contact electrode work function was determined as discussed in section "Effect of back contact electrode on the proposed inverted perovskite solar cell". The characteristics of the device’s material parameters adopted were carefully selected from theories, experiments and research reviews is presented in Table 1, while the interface parameters are presented in Table 2. Scientifically, the neutral defect type adopted in the simulation means non-reactive, which can further be explained as a situation where there is no donor nor acceptor of charges within the films of a layer or interface. The bulk defect densities of the materials were chosen above ideal values to demonstrate ideal experimental conditions.

**Table 1:** Parameters for modeling and simulation of an inverted planar perovskite solar cell structure using CH_3_NH_3_SnI_3_, CU_2_O and ZnO as Absorber, HTM and ETM respectively.

Parameter	CU_2_O (HTM)	CH_3_NH_3_SnI_3_ (Absorber)	ZnO (ETM)
Thickness (nm)	50	400	50
Bandgap energy Eg (eV)	2.17^61^	1.3^62^	3.3^63^
Electron affinity χ (eV)	3.2^61^	4.17^62^	4.0^63^
Relative Permittivity $\in_r$	7.5^63^	6.5^63^	9.0^63^
Effective conduction band density CB (1/cm^3^)	2.0 × 10^18^^63^	1.0 × 10^18^^63^	2 × 10^18^^63^
Effective valence band density VB (1/cm^3^)	1.8 × 10^18^^63^	1.0 × 10^19^^63^	1.8 × 10^19^^63^
Electron thermal velocity (cm/s)	1 × 10^07^^61^	1 × 10^07^^62^	1 × 10^07^^36^
Hole thermal velocity (cm/s)	1 × 10^07^^61^	1 × 10^07^^62^	1 × 10^07^^36^
Electron mobility µn (cm^2^/Vs)	20^63^	2000^60^	100^63^
Hole mobility µp (cm^2^/Vs)	80^63^	300^60^	25^63^
Shallow uniform donor density ND (1/cm^3^)	0.0^61^	0.0^61^	1.0 × 10^18^^63^
Shallow uniform acceptor density NA (1/cm^3^)	2.0 × 10^19^^63^	3.0 × 10^17^^62^	0.0^36^
Defect type	Neutral^61^	Neutral^62^	Neutral^36^
Capture cross section hole (cm^2^)	1 × 10^–15^^61^	1 × 10^–15^^62^	1 × 10^–15^^36^
Capture cross section electrons (cm^2^)	1 × 10^–15^^61^	1 × 10^–15^^62^	1 × 10^–15^^36^
Energetic distribution	Single^61^	Gaussian^61^	Single^36^
Reference for defect energy level Et	Above Ev^61^	Above Ev^62^	Above Ev^36^
Energy level with respect to Reference (eV)	0.600^61^	0.600^62^	0.600^36^
Defect density N_t_ (1/cm^3^) uniform	1.0 × 10^14^^63^	2.0 × 10^15^^63^	1.0 × 10^15^^63^

**Table 2:** Parameters of interface layer.

Interlayer	Cu_2_O/ CH_3_NH_3_SnI_3_	CH_3_NH_3_SnI_3_/ZnO
Defect type	Neutral	Neutral
Capture cross section hole (cm^2^)	1 × 10^–19^	1 × 10^–19^
Capture cross section electrons (cm^2^)	1 × 10^–19^	1 × 10^–19^
Energetic distribution	Single	Single
Reference for defect energy level Et	Above the highest Ev	Above the highest Ev
Energy with respect to reference (eV)	0.600	0.600
Total density (integrated over all energies) (1/cm^3^)	1 × 10^10^	1 × 10^10^

Various decisive parameters like electron mobility, hole mobility, carrier diffusion length, interfacial resistance, etc., have been considered constant and taken from the literature. These parameters are extremely dependent on experimental processes and can hugely alter practical performance of the device. The relative humidity, temperature, the type of instruments used, procedural and human expertise, control of crystallization and grain growth rates are some of the factors behind the real-life performance and their variations from theoretical values.

## Results and discussions

**Figure 1:**
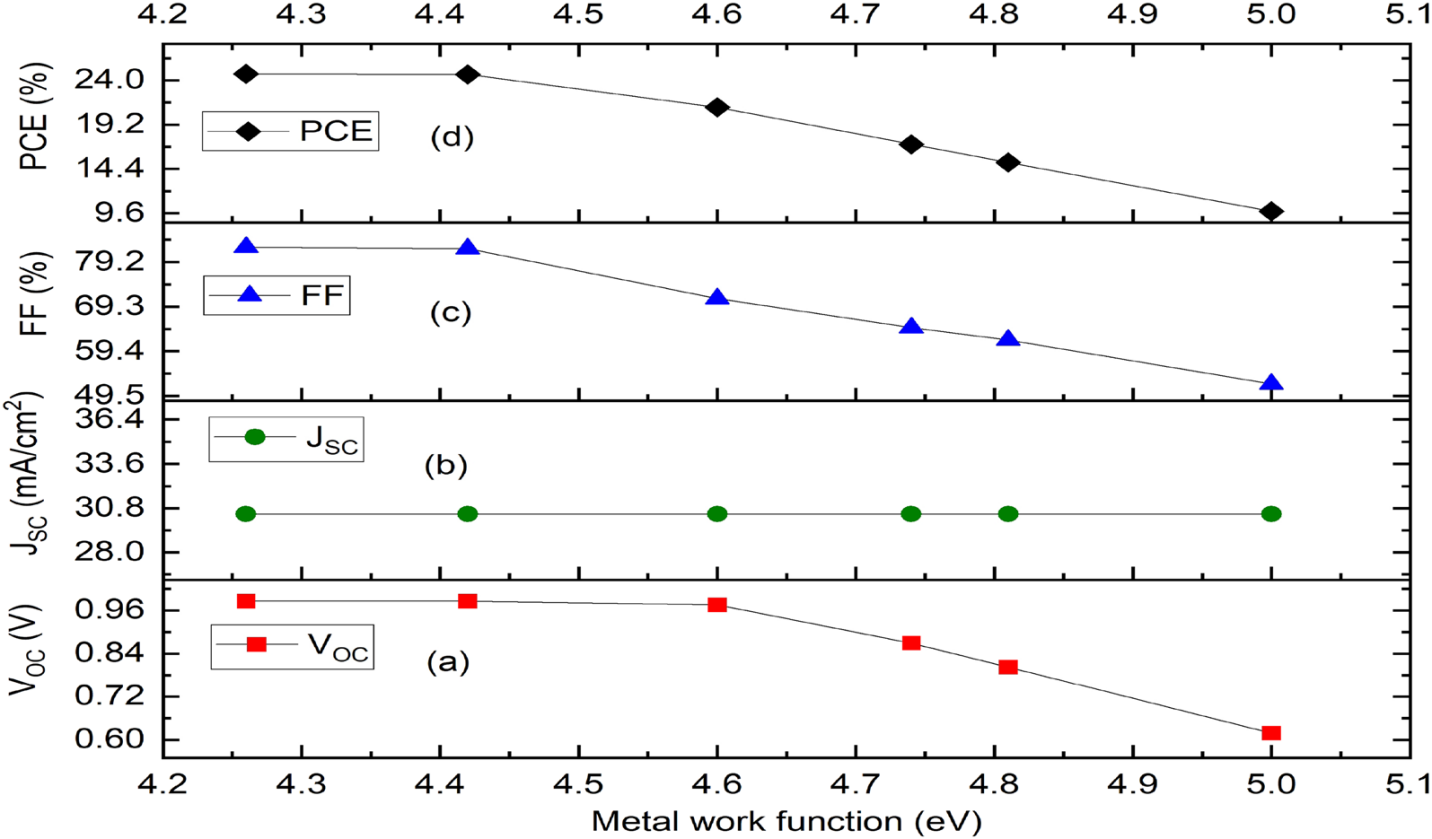
Effect of different back metal contact electrodes on parameters of the proposed IPSC. (**a**) Plot of V_OC_ against metal work function, (**b**) Plot of J_SC_ against metal work function, (**c**) Plot of FF against metal work function, (**d**) Plot of PCE against metal work function.

In general, the electron and hole pairs are produced within the absorber layer after illumination. The junction field causes holes and electrons to travel in the directions of HTM and ETM layers, respectively. A voltage is created when these holes and electrons are collected at the anode and cathode, respectively. The simulation results of the proposed inverted device structure Cu_2_O/CH_3_NH_3_SnI_3_/ZnO using the available initial device parameters as contained in Tables 1 and 2 shows the J–V characteristics of the proposed device as shown in Fig. 1 produced a Voc of 0.9854 V, J_SC_ of 30.4185 mA/cm^2^, an FF of 82.48% and PCE of 24.72%. The proposed device structure Cu_2_O/MASnI_3_/ZnO underwent further simulation and optimization so as to obtain optimized thickness of the constituent layers.

### Effect of back contact electrode on the proposed inverted perovskite solar cell

**Figure 2:**
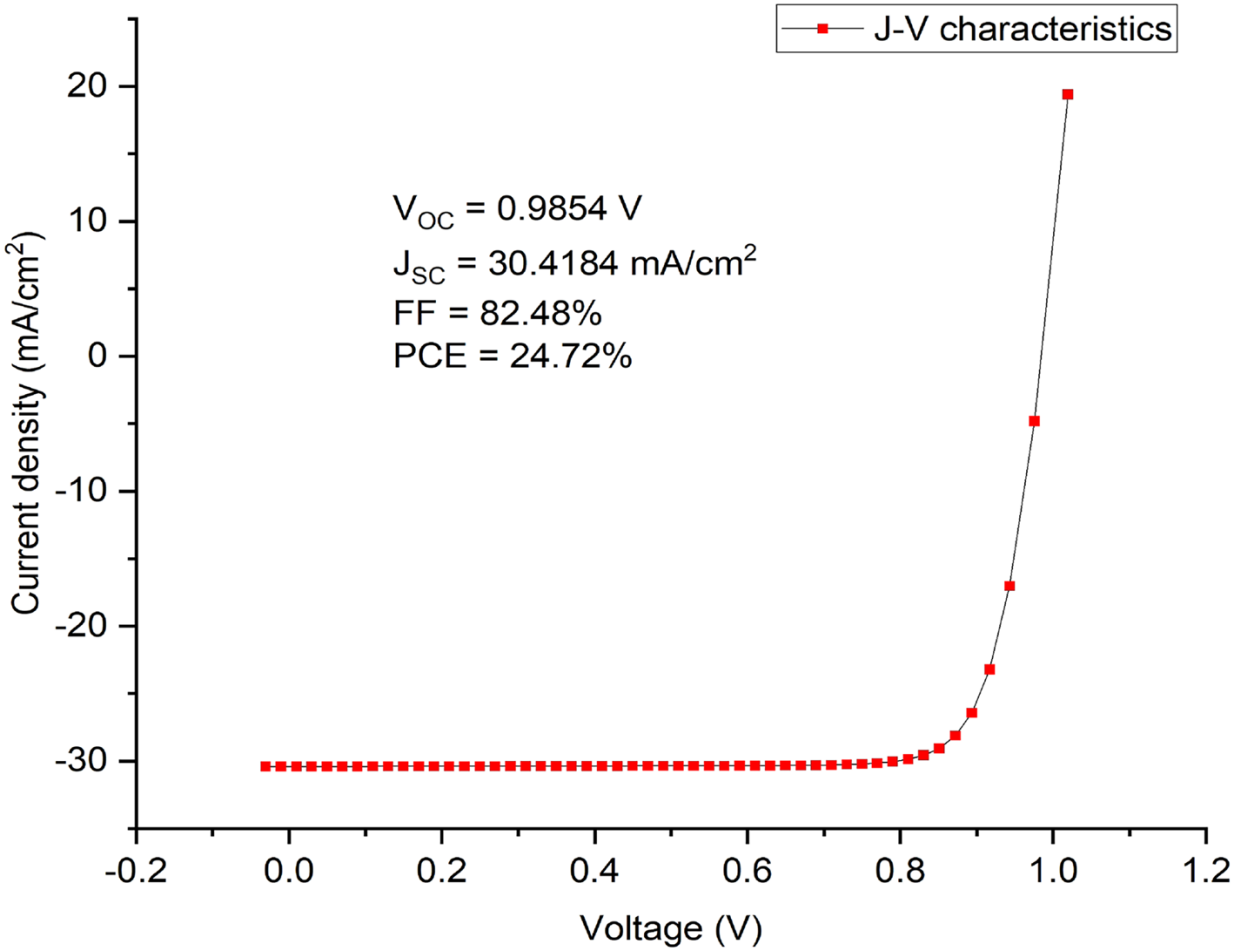
J–V Characteristics of the proposed inverted perovskite solar cell with initial parameters using MASnI_3_ as absorber material, Cu_2_O as HTM, ZnO as ETM and Al as back contact respectively.

Various metal back contact electrodes such as aluminium (4.26 eV), tin (4.42 eV) graphene (4.60 eV), silver (4.74 eV), iron (4.81 eV) and copper (5.00 eV) have been tested on the proposed inverted structure so as to determine the most appropriate one to be used for enhanced optimal performance. Figure 1 shows the work function of various metals used as back contact electrodes and their associated photovoltaic parameters on the proposed IPSC based device simulated using initial given parameters presented in Tables 1 and 2. The results in Fig. 1 clearly show that the choice of aluminum (Al) for back electrode maintained the most optimal device performance, as the V_oc_, J_SC_, FF and PCE of 0.9854 V, 30.4185 mA/cm^2^, 82.48% and 24.72% respectively is produced. It is interesting to note in this model that the J_SC_ (Fig. 1b) remains constant as the work function of the back contact varies while the V_OC_, FF and PCE declines as the work function increases from 4.26 to 5.00 eV (Fig. 1a,c,d). For p–n configuration, the current is negative because of the uphill diffusion of the minority charge carriers in terms of concentration gradient arising from reverse bias during solar illumination. The current growth from the negative quadrant towards the positive quadrant signifies power generation up to zero value of current where an open circuit voltage (V_OC_) of 0.9854 V is achieved. The J–V characteristics of the device having used aluminum as the back contact electrode is shown as Fig. 2.

### Effect of n-type TCO-free on inverted perovskite solar cell architecture.

There is no experimental result for this exact structure (Cu_2_O/CH_3_NH_3_SnI_3_/ZnO/Al) known to us, which makes this research novel and interesting. There is no clear reasons why the lack of experimental works to support this study, but this could be due to lack of good conductivity of all-inorganic transport materials in nano electronics compared to organic transport materials and high processing temperature required. However, there are few simulation results of exact combination in n–i–p structure reported in^63,64^. The PCEs of 26.55% and 9.27% respectively were obtained in^63^ and^64^, while our designed n-type TCO-free p-i-n device produced a superior PCE of 30.17% as shown in Fig. 8.

The proposed inverted model will not simulate when the conventional n-type TCO (ITO/FTO) of donor concentration ND is used with the acceptor concentration NA being zero, except an organic p-type TCO is used which is outside the scope of this study. It’s worth noting that the top transparent glass used as presented in Fig. 16 is an n-type TCO-free substrate in order to avoid non-convergence of voltage between the front and back electrodes when a conventional n-type TCOs are used. The carefully chosen transparent glass substrate size of 50 nm is not included in the simulation model, hence it is undoped and may not have significant impact on the device in real experimental situation. In this work, it is difficult to drive an output from the device if a TCO of an n-type material (FTO/ITO) is used as front electrode in the inverted structure when the same n-type material of same polarity is used as back hole blocker (ZnO) because of non-voltage convergence arising from non-compatible work function between the layers. The non-voltage convergence experienced when ITO with metal function of 4.7 eV is used is as a result of non-ideal band gap between the adjacent semiconductors layers (ITO/Cu_2_O) which makes the proposed n-type TCO-free model feasible. However, a back contact electrode of low metal function lower than ZnO like aluminium (4.26 eV) is required for optimal performance.

The use of Cu_2_O as front contact electrode may suffer setback due its high sheet resistance and poor conductivity when compared to n-type TCOs. However, the sheet resistance of most metal oxides depends on the method of deposition, temperature, oxygen flow rate and thickness of the films. The control of power and oxygen flow rates during deposition of copper oxide thin films at a thickness of less than 100 nm prepared by reactive magnetron sputtering can reduce the sheet resistance and enhance performance of the device in practical sense^65^. The provision of a high density of low energy sputtered copper radicals/ions, and when combined with a controlled amount of oxygen, can produce good quality p-type transparent Cu_2_O films with electrical resistivity ranging from 10^2^ to 10^4^ Ω-cm^66^ which makes Cu_2_O a potential transparent front conducting oxide for photovoltaic applications. Also, the doping of Cu_2_O with nickel can improve its p–type conductivity via extrinsic doping and post–growth processing^67^. Therefore, the Cu_2_O may not be as conductive as other n-type TCOs in experimental sense but runs conveniently in the simulation model without challenge which means the proposed n-type TCO–free model is novel and less complex, providing good direction in the design and modeling of simple inverted perovskite solar cells as shown in Figs. 13 and 16. Cu_2_O can act as a front electrode efficiently provided its thickness is thin enough to ensure adequate clarity and transparency to enhance admittance of photons into the absorber (perovskite) layer.

### Effect of thickness of the HTM (Cu_2_O), absorber (CH_3_NH_3_SnI_3_) and ETM (ZnO) layers

**Figure 3:**
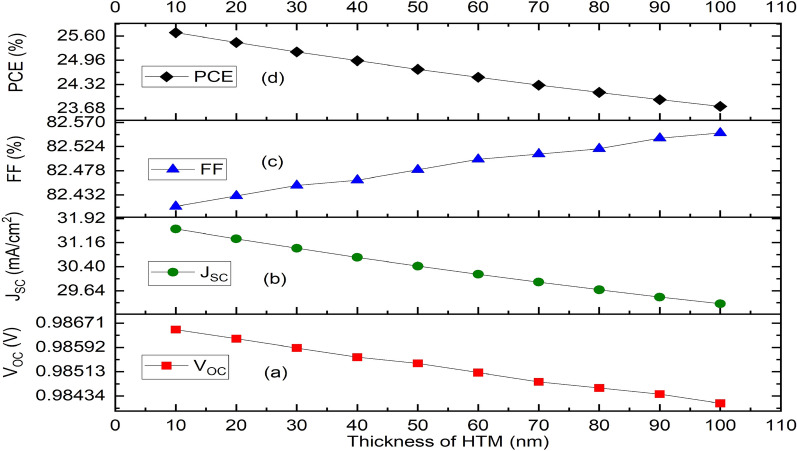
Effect of variation of thickness of the HTM layer (Cu_2_O) on solar cell parameters. (**a**) Plot of V_OC_ against thickness, (**b**) Plot of J_SC_ against thickness, (**c**) Plot of FF against thickness, (**d**) Plot of PCE against thickness.

In this study, the variation of HTM’s layer thickness from 10 to 100 nm results to a slight increase in FF (Fig. 3c) while a decline in device parameters such as V_OC_, J_SC_ and PCE is experienced as presented in Fig. 3a,b,d respectively.

**Figure 4:**
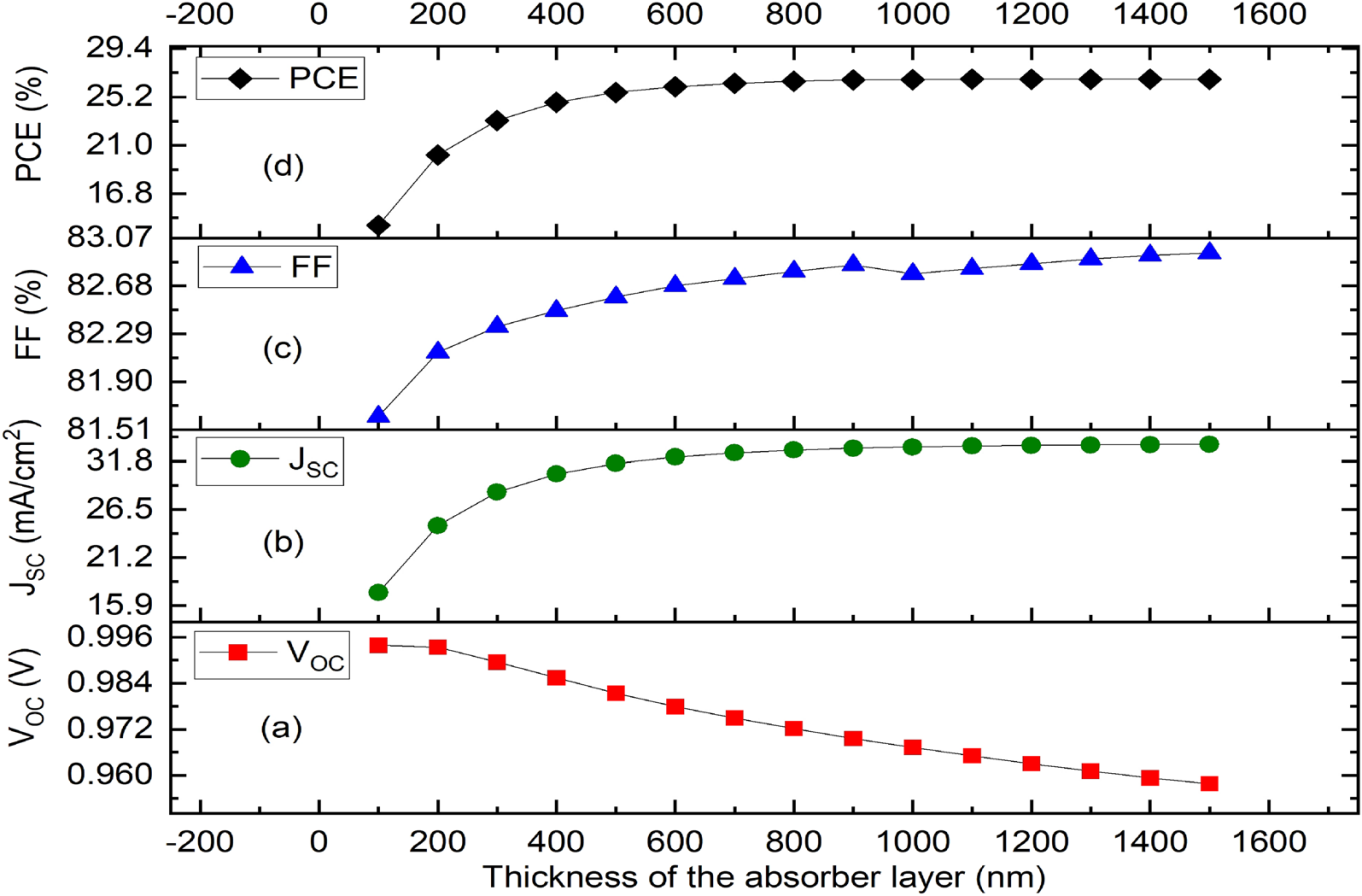
Effect of variation of thickness of the absorber layer (MASnI_3_) on solar cell parameters. (**a**) Plot of V_OC_ against thickness, (**b**) Plot of J_SC_ against thickness, (**c**) Plot of FF against thickness, (**d**) Plot of PCE against thickness.

The thickness of the absorber layer considerably affects the solar cell’s overall performance. The increase in absorber’s thickness decreases the V_OC_ due to increase in series resistance. Meanwhile, the increase in absorber’s thickness increases the J_SC,_ FF and PCE to the maximum after which it decreases with further increase in thickness. In this study, the thickness of CH_3_NH_3_SnI_3_ has been adjusted in this simulation from 100 to 1500 nm. The fluctuation of photovoltaic characteristics with thickness of absorber layer is shown in Fig. 4. The V_OC_ declines as a result of faster recombination due to increased thickness (Fig. 4a). A thicker absorber layer absorbs more photons, which increases short circuit current density (J_CS_) and the fill factor (FF) and as seen in Fig. 4b,c, respectively. The solar cell efficiency is increased as the thickness of absorber layer increases up to an ideal thickness for the solar cell after which efficiency declines (Fig. 4d). However, as diffusion necessitates a longer charge travel distance, recombination is more common in larger absorber layers; hence, efficiency decreases after a certain thickness value. Our results concur with experimental findings in^62,68^. As shown in Fig. 4d, the ideal absorber layer thickness for this inverted PSC is achieved between 1200 and 1300 nm.

**Figure 5:**
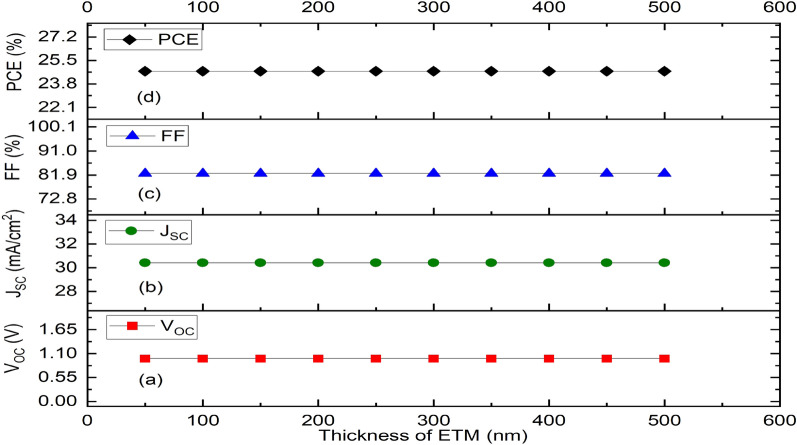
Effect of variation of thickness of the ETM layer (ZnO) on solar cell parameters. (**a**) Plot of V_OC_ against thickness, (**b**) Plot of J_SC_ against thickness, (**c**) Plot of FF against thickness, (**d**) Plot of PCE against thickness.

Nevertheless, the increase in ETM’s thickness leads to a non-noticeable change in V_OC_, J_SC_, FF and PCE (Fig. 5a–d) respectively. Therefore, it can be inferred that while device performance is mostly determined by absorber thickness, IPSC device performance is not influenced by the ETM layer’s thickness but rather varies slightly with the HTM’s thickness, which is designed to be small enough to guarantee optical transparency and ensure easy photon penetration to the absorber layer. The selection of optimal thickness is important to regulate series and shunt resistance and ensure improved device performance in terms of short circuit current, open circuit voltage, fill factor and power conversion efficiency.

### Simulation and thickness optimization of the proposed device structure

**Figure 6:**
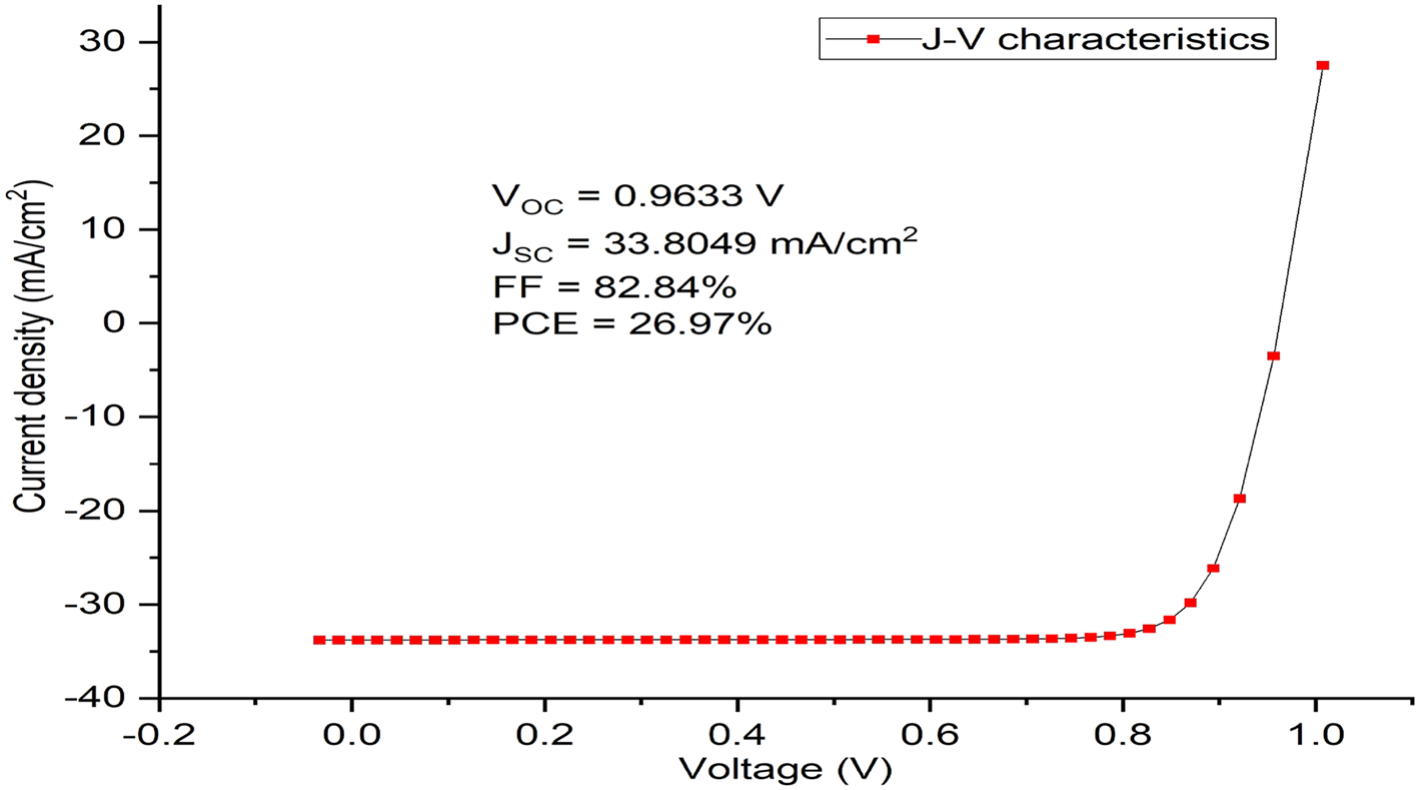
J–V characteristics of the optimized device’s thickness using MASnI_3_ as absorber material, Cu_2_O as HTM, ZnO as ETM and Al as back contact respectively.

Simulation and optimization of the proposed device shows that the HTM (Cu_2_O) layer, the absorber (MASnI_3_) layer and ETM layer (ZnO) have been optimized to the thickness of 40 nm, 1200 nm and 200 nm respectively. The simulation of these optimized dimensions led to an improvement in the solar cell parameters as it produced a Voc of 0.9633 V, J_SC_ of 33.8049 mA/cm^2^, FF of 82.84% and PCE of 26.97% as shown in the J–V characteristics curve (Fig. 6).

### Effect of absorber’s doping concentration (NA)

**Figure 7:**
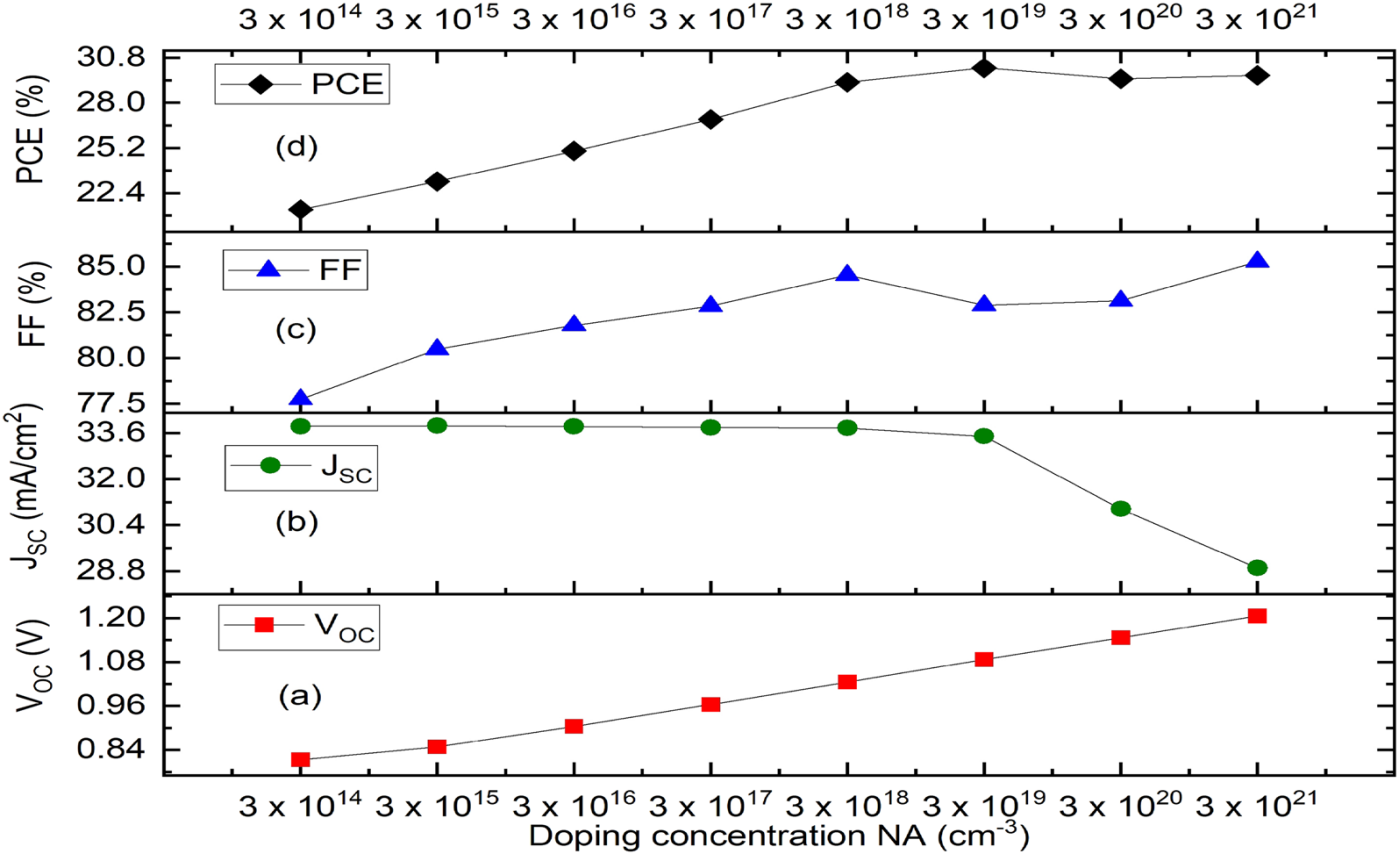
Effect of variation of doping concentration of the absorber (CH_3_NH_3_SnI_3_) on solar cell parameters. (**a**) Plot of V_OC_ against doping concentration (NA), (**b**) Plot of J_SC_ against doping concentration (NA), (**c**) Plot of FF against doping concentration (NA), (**d**) Plot of PCE against doping concentration (NA).

**Figure 8:**
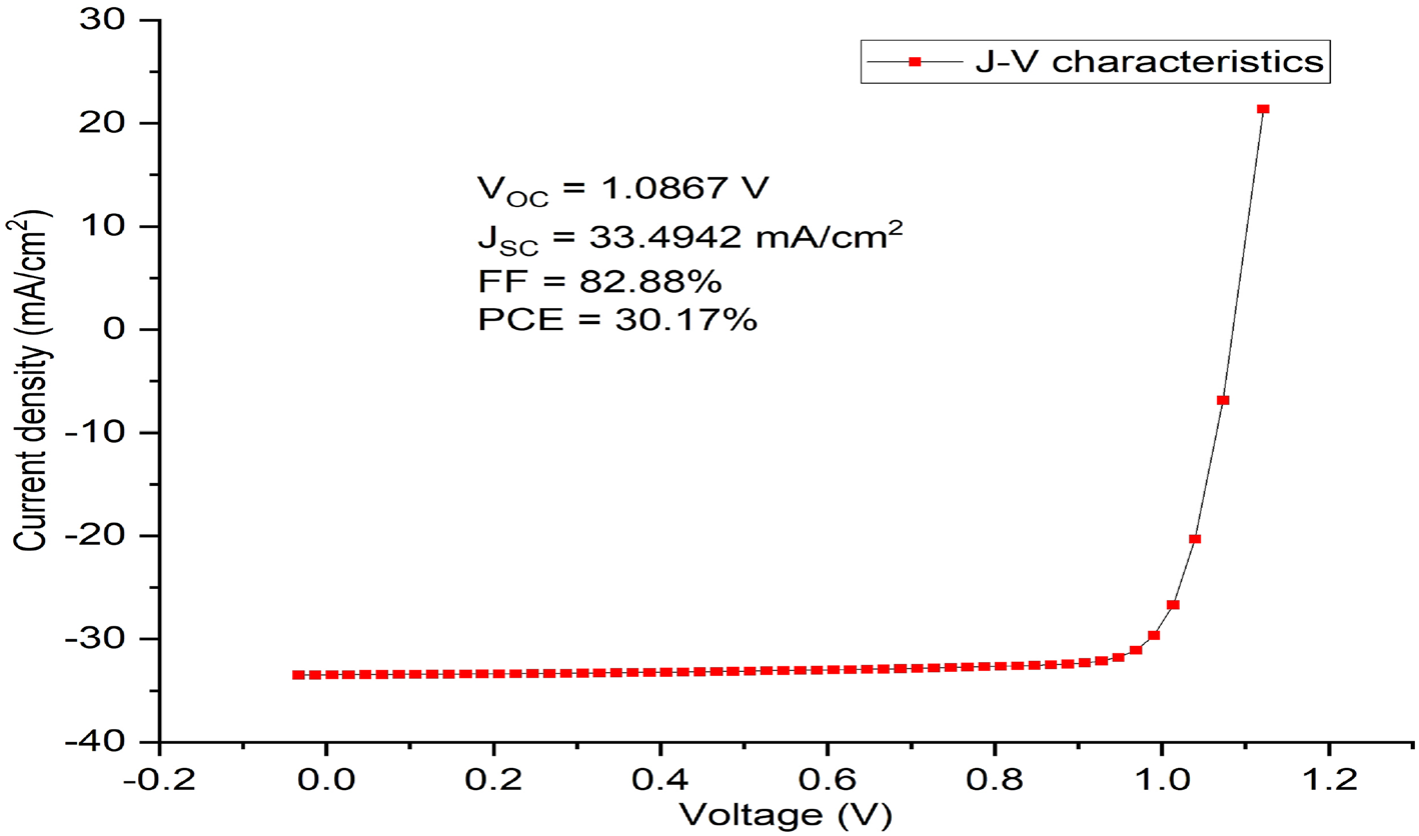
J–V Characteristics of the final optimized inverted simulated solar cell device using MASnI_3_ as absorber material, Cu_2_O as HTM, ZnO as ETM and Al as back contact respectively.

The holes’ acceptor density of the absorber layer has a major impact on the photovoltaic cell’s device performance in addition to its thickness. As demonstrated in Fig. 7, the Fermi energy level of the hole falls with increasing doping concentration of the acceptor, and as a result, V_OC_ increases (Fig. 7a). Also, an increase in the doping concentration of the acceptor leads to a built-in potential that increases charge separation, which in turn causes a rise in V_OC_. In this work, the acceptor concentration NA (1/cm^3^) of the absorber layer is varied within a range of 3 × 10^14^ cm^−3^ to 3 × 10^21^ cm^−3^ to ascertain the most optimal value that can produce an optimal performance of the proposed device. Nevertheless, J_SC_ maintains a steady decline marginally up to NA’s value of 3 × 10^19^ cm^−3^ before falling off sharply. At the same NA’s value, the value of FF drops suddenly which might be caused by a rise in the rate at which charge carriers within the absorber layer recombine or an increase in series resistance^55^. The absorber layer’s doping concentration value of 3 × 10^19^ cm^−3^ produced the best cell performance having V_oc_ of 1.0867 V, J_SC_ of 33.4942 mA/cm^2^, FF of 82.88% and PCE of 30.17% as shown in Fig. 7a–d respectively, while its J–V characteristics is shown as Fig. 8.

The complex nature of an organic molecule in the A site of the perovskite structure (ABX_3_) may be the cause of degradation, as evidenced by the absorber’s bandgap of 1.3 eV and the measured Voc of 1.0867 V. Using varying ratios of the precursors causes an intrinsic fault when the perovskite structure is distorted. Higher degrees of crystallization and a slower rate of breakdown are the results of vacancies in the structure caused by the excess CH_3_NH_3_I (MAI). The crystalline lattice’s anomalies emphasize the role MAI plays in the deterioration process. Excess MAI may potentially release halide ions, depending on the concentration. Afterwards, these halide ions function as dopants, altering the perovskite semiconductors' bandgap^69^. When exposed to air, the Sn^2+^ in CH_3_NH_3_SnI_3_ is changed to Sn^4+^ (a process known as self-doping), converting the device into a p-type semiconductor. Sadly, this procedure deteriorates the device performance, such as the output power and the power conversion efficiency^56,70^.

### Effect of series resistance R_series_ and shunt resistance R_shunt_

The resistance in series and shunt (R_series_ and R_shunt_) affects the J–V curve’s form and slope, which in turn affects the solar cell’s efficiency. The connections electrodes, electrical dissipation in the perovskite, and layers of hole and electron transport materials (HTM and ETM) are primarily linked to the cause of the R_series_. However, different recombination pathways, device design, and defects induced during the layer deposition process are linked to the cause of the R_shunt_. According to the literature, a high shunt resistance and a low series resistance are necessary for a solar cell to have a high efficiency. Electrons cannot flow freely across a circuit if the series resistance is large, and leakage current will occur if the shunt resistance is low, producing PSCs with low stability and efficiency. When there’s a low shunt resistance or a high series resistance, the PSC’s maximum output and FF would both drop^71,72^. The ideal diode model’s Eq. (4) was applied in order to comprehend the impact of R_series_ and R_shunt_ on the perovskite solar cell’s performance^73^.

4 $$J={J}_{L}-{J}_{O}\left[{e}^{\frac{\left[q(V+{J}^{*} {R}_{series}\right]}{AkT}}-1\right]-\frac{V+ {J}^{*} {R}_{series}}{{R}_{shunt}}$$

When $J\approx$ 0 mA/cm^2^ for open circuit state, the variables V_OC_ and R_shunt_ relationship is presented in Eq. (5)

5 $${R}_{shunt}=\frac{{V}_{OC}}{{J}_{L}-{J}_{O}\left[{e}^{\frac{\left[q({V}_{OC}\right]}{AkT}}-1\right]}$$

where J is the current flowing via the external circuit, V is the output voltage, A is the ideality factor, k is the Boltzmann constant, T is the temperature, q is the electron charge, J_O_ is the saturation current density and J_L_ is the light-induced current density. As a result, low R_shunt_ reduces photovoltaic voltage and may also have an impact on the photocurrent that is collected, whereas high R_series_ values primarily influence the FF and Jsc values^72^.

**Figure 9:**
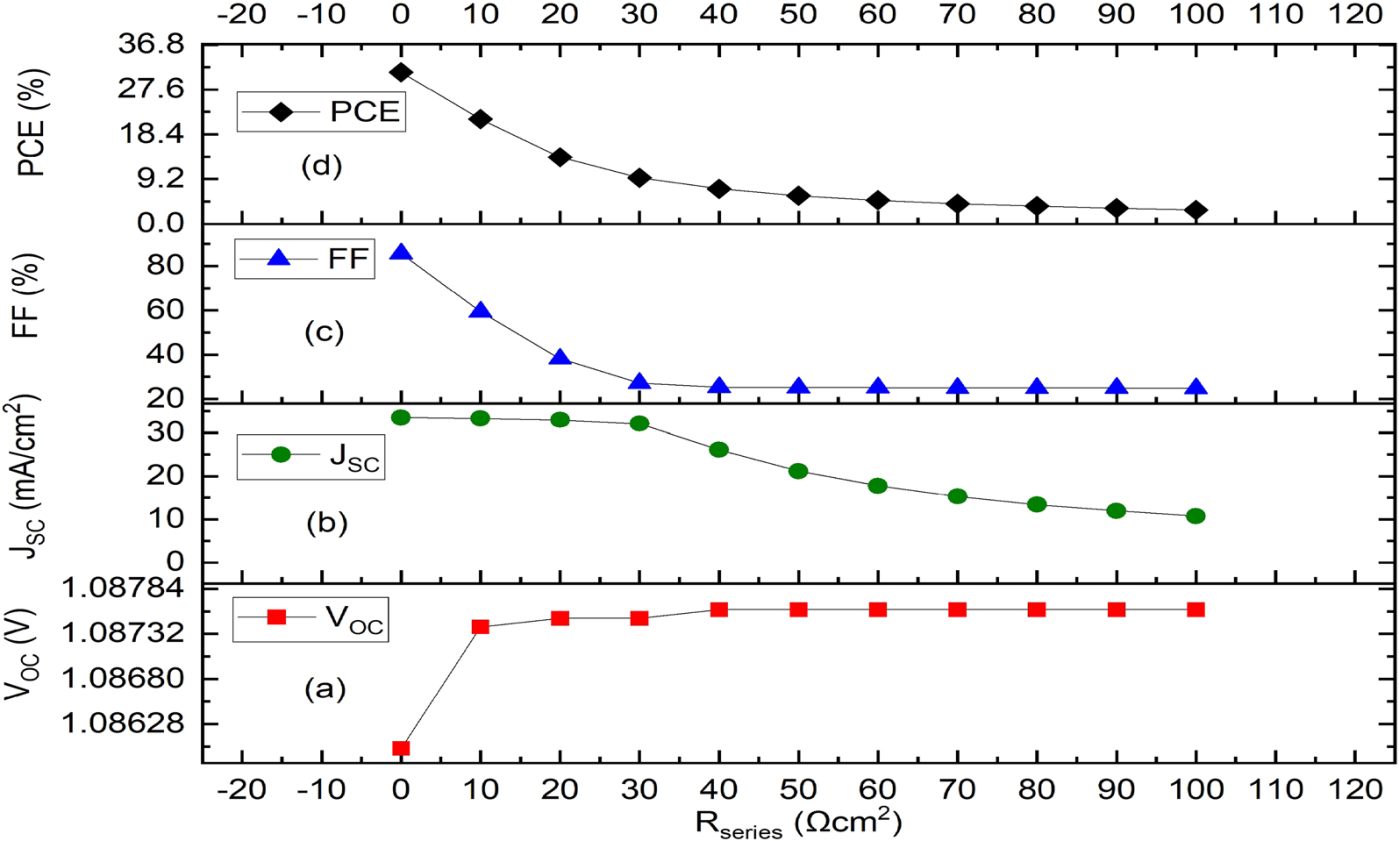
Effect of series resistance variation on the optimized IPSC based device parameters. (**a**) Plot of V_OC_ against series resistance. (**b**) Plot of J_SC_ against series resistance. (**c**) Plot of FF against series resistance, (**d**) Plot of PCE against series resistance.

**Figure 10:**
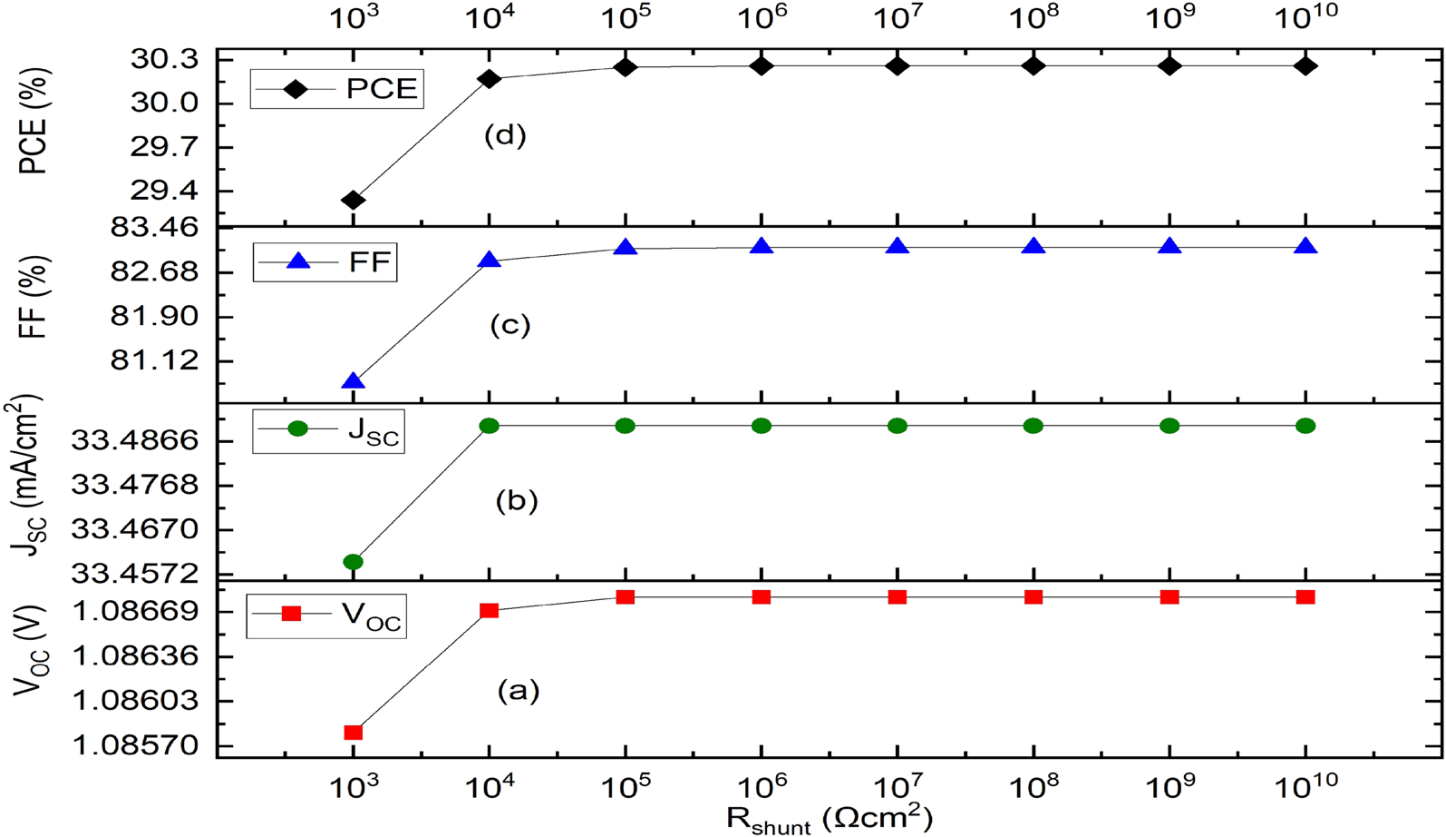
Effect of shunt resistance variation on parameters of the optimized IPSC device. (**a**) Plot of V_OC_ against shunt resistance, (**b**) Plot of J_SC_ against shunt resistance, (**c**) Plot of FF against shunt resistance, (**d**) Plot of PCE against shunt resistance.

While keeping the other simulation parameters same, R_series_ and R_shunt_ were changed from 0 to 100 Ωcm^2^ and 10^3^ Ωcm^2^ to 10^10^ Ωcm^2^ respectively, to better understand their influence on the J–V curves. The responses of V_OC_, J_SC_ FF and PCE as a function of R_series_ are presented in Fig. 9. V_OC_ stays fairly constant, J_SC_ falls from 33.51 to 10.77 mA/cm^2^, and FF drops from 85.63 to 24.88% while R_series_ grows from 0 to 100 Ωcm^2^. As a result, as Fig. 9d illustrates, PCE’s behavior is precisely proportional to J_SC_ and FF, decreasing from 31.16 to 2.91% for the same range. Alternatively, as Fig. 10 illustrates, when R_shunt_ rises from 10^3^ to 10^10^ Ωcm^2^, V_OC_ rises from 1.0858 to 1.0868 V, J_SC_ maintains a constant 33.49 mA/cm^2^ from 10^4^ Ωcm^2^, FF rises from 80.75 to 83.12%, and the PCE rises from 29.34 to 30.26% respectively (Fig. 10a–d). For R_series_ and R_shunt_, the optimal values are therefore 1 Ωcm^2^ and 10^6^ Ωcm^2^ respectively, which is in conformity with literature.

### Effect of the defect state of bulk and interface layers

The impact of the absorber’s defect density is an important factor that needs to be examined. In the absorber layer, defects are inevitable. Both at surfaces and in the bulk, they are present. Point defects in the perovskite absorber layer include lattice vacancies, interstitial, Schottky, and Frenkel defects. In addition, there may be higher order defects like grain boundaries and dislocations^74^. The self-doping process in the absorber layer creates the p-type semiconductor that results in an impurity defect^54,56,75,76^. These defects cause the energy bandgap to appear at shallow or deep levels^74^. Charge carriers have the ability to capture and promote nonradiative recombination of electron–hole as a result of these defects^53,55^. Noteworthy, the simulated interface defect density for both electron and hole recombination velocities was 1 × 10^-2^ cm/s for both HTM/MASnI_3_ and ETM/MASnI_3_ interface. In the Sn-based perovskite absorber layer, the electron and hole diffusion lengths were 16 µm and 6.2 µm, respectively. The optimized device’s absorber defect density (Nt) of 2 × 10^15^ cm^−3^ achieved a V_OC_ of 1.0867 V, a J_SC_ of 33.4942 mA/cm^2^, FF of 82.88%, and a PCE of 30.17%. Nevertheless, synthesizing a material with a low defect density value is a challenging task in an experiment^55^.

**Figure 11:**
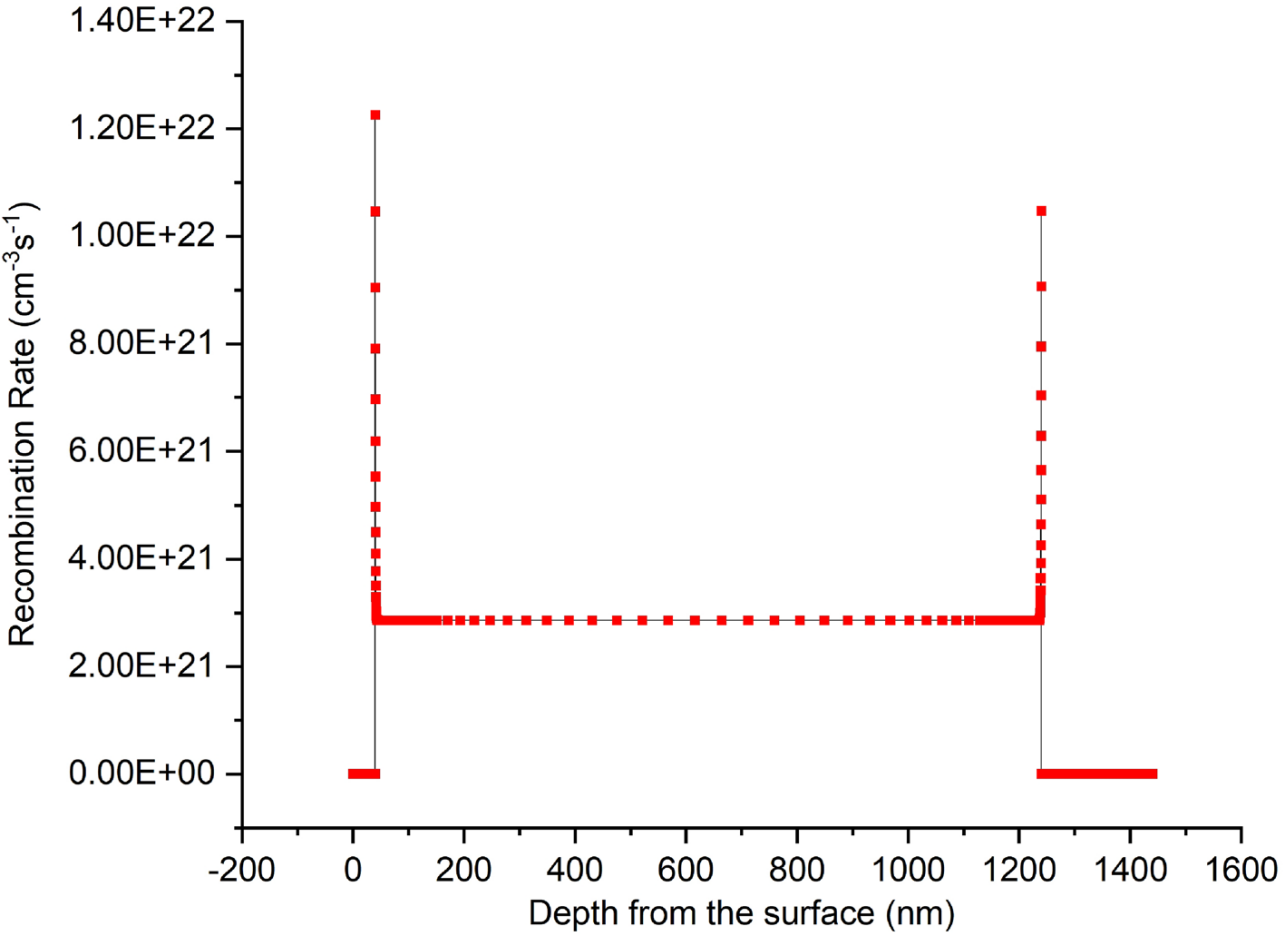
Recombination rate of the optimized device with depth from the surface.

The Shockley–Read–Hall (SRH) recombination model has been applied to provide understanding regarding the impact of defect density in the absorber layer on device performance^49,53,77^. The effect of defect density on the recombination rate based on the SRH recombination model is essential to determining the critical influence of Nt on the device performance. The plot of recombination rate with depth from the optimized device’s surface is depicted in Fig. 11.

**Figure 12:**
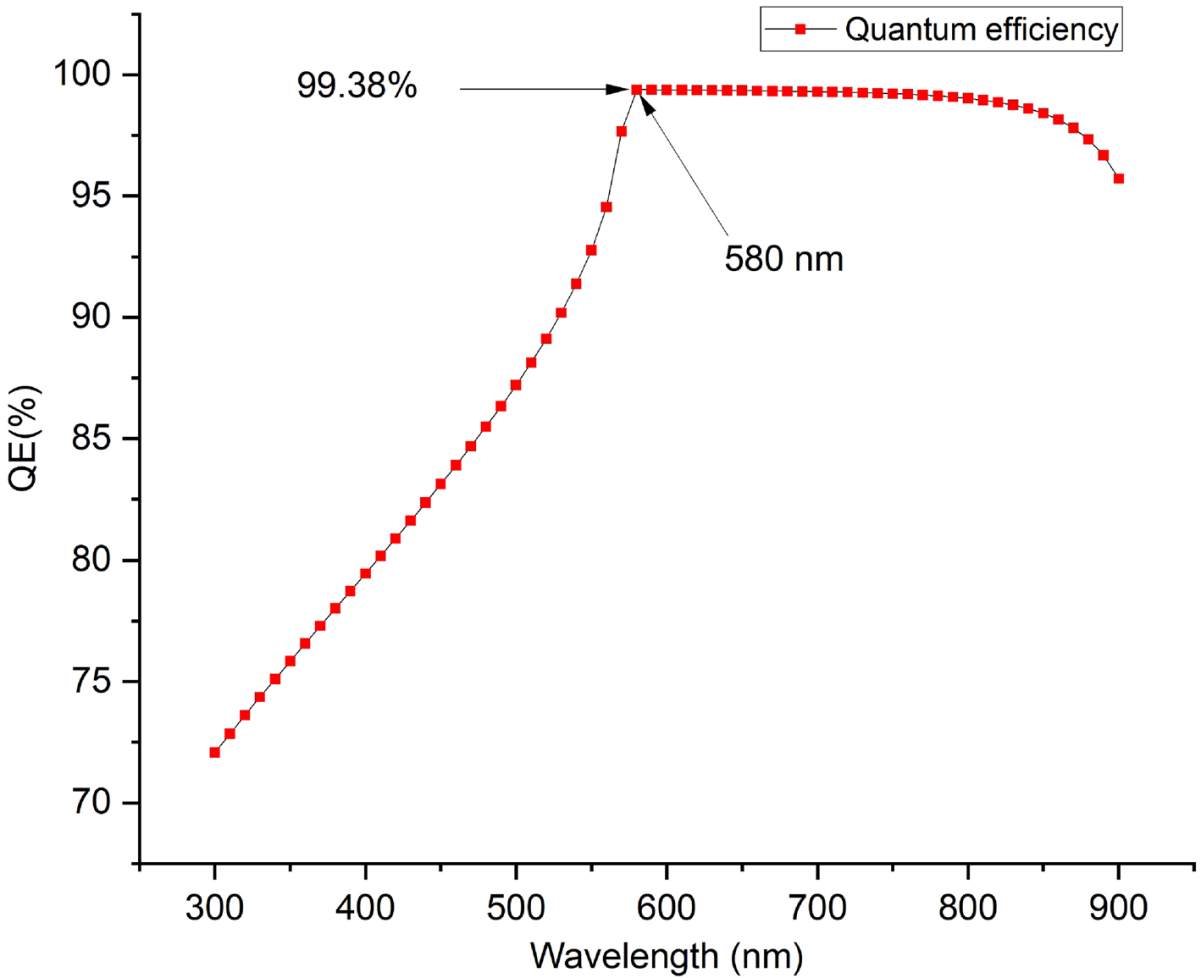
Quantum efficiency of the proposed inverted perovskite solar cell.

**Figure 13:**
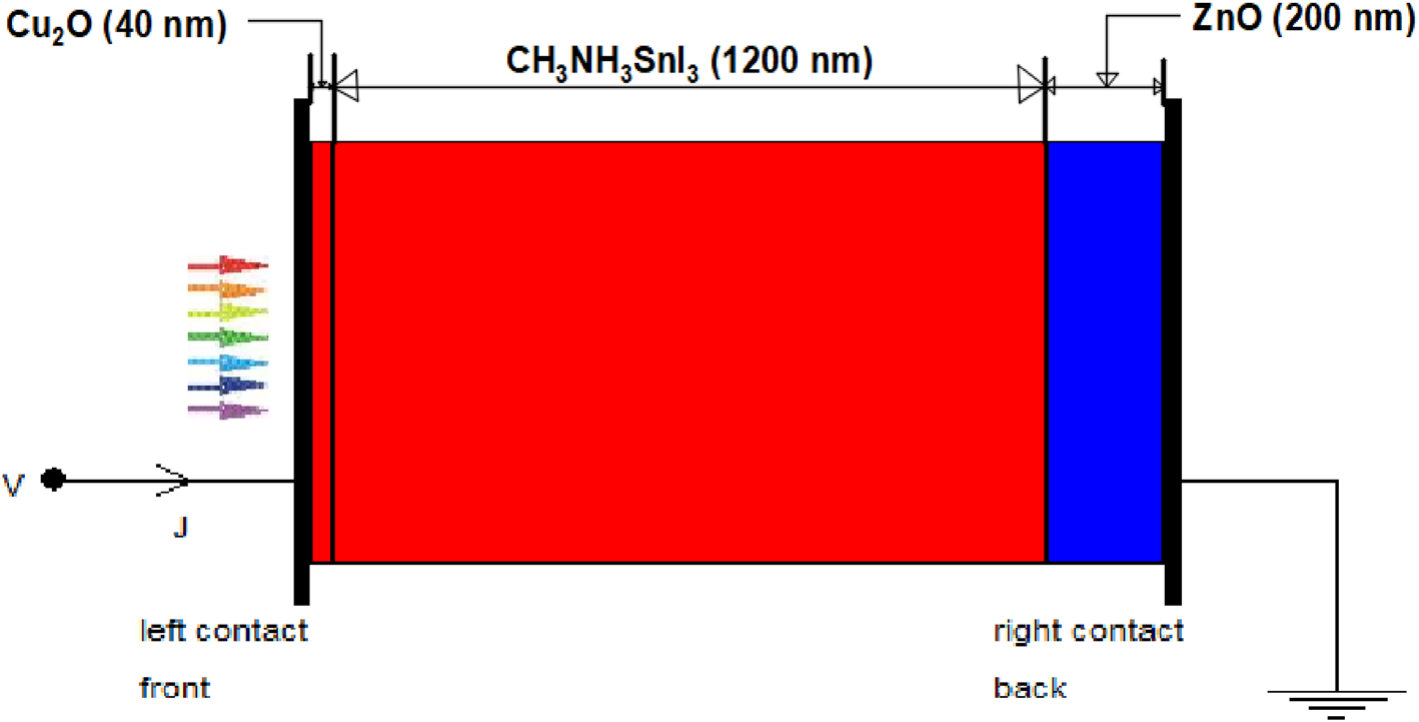
The simulated inverted device structure.

**Figure 14:**
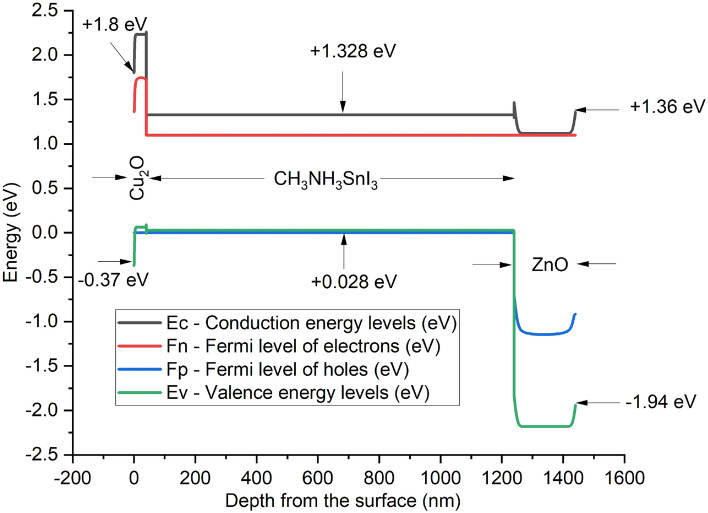
Energy band diagram of the proposed inverted perovskite solar cell.

**Figure 15:**
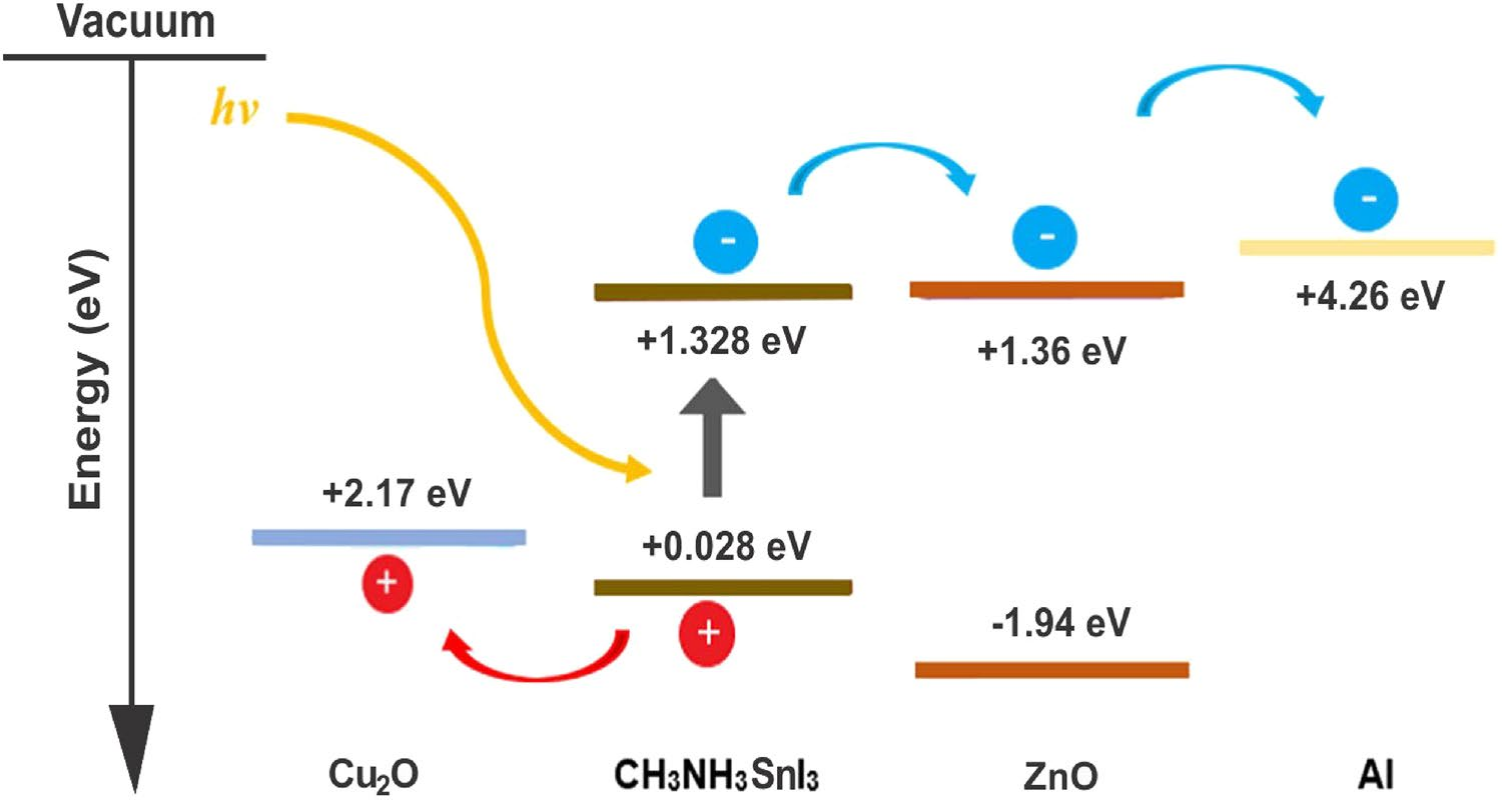
Energy band alignment profile of the proposed inverted perovskite solar cell.

**Figure 16:**
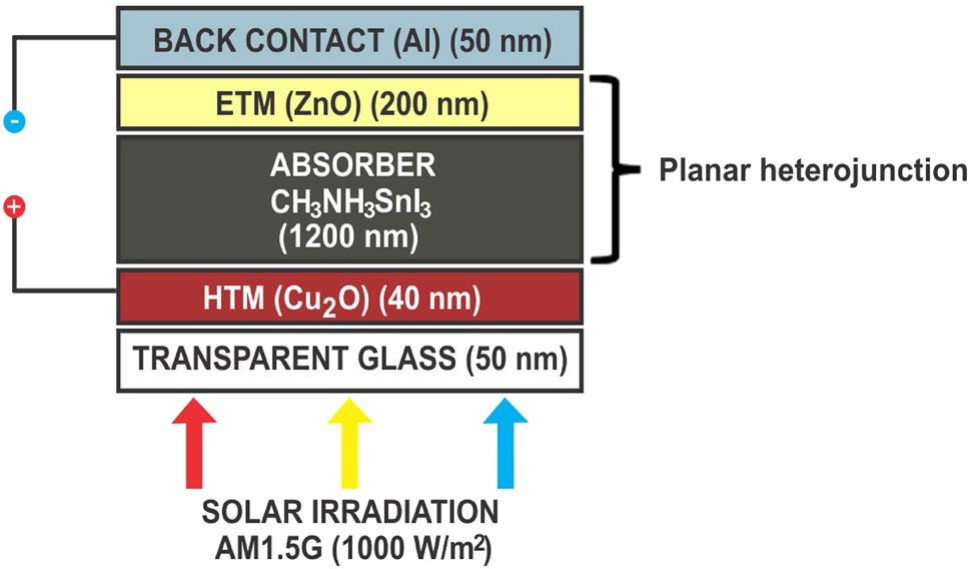
The complete optimized structure of the proposed n-type TCO-free inverted perovskite solar cell.

The proposed device produced quantum efficiency curve covering the entire visible spectrum (300–900 nm) achieving an optimum quantum efficiency (QE) of 99.38% at 580 nm wavelength, which is in agreement with other works^15,43,54,61,78,79^ is presented as Fig. 12. The simulated inverted structure, energy band diagram, energy band alignment and complete device structure of the optimized inverted planar perovskite solar cells are presented as Figs. 13, 14, 15 and 16, respectively. It’s very clear that the photovoltaic performance of the proposed device as shown in Table 3 is superior to other related works reported in the literature.

**Table 3:** Photovoltaic parameters of either Cu, Zn or Sn-based perovskite solar cells of some reported experimental and simulated works from the literature.

Device structure	Parameters				Study type	References
HTM/Absorber/ETM	V_OC_ (V)	J_SC_ (mA/cm^2^)	FF (%)	PCE (%)		
Cu_2_O/CH_3_NH_3_SnI_3_/ZnO	0.850	32.26	74.02	20.23	Simulation	^80^
CuO/CH_3_NH_3_SnI_3_/ZnO	0.830	29.71	61.23	15.10	Simulation	^81^
HTM-free /CH_3_NH_3_SnI_3_/C_60_	0.858	32.45	73.72	20.54	Simulation	^82^
Cu_2_O/CH_3_NH_3_PbI_3_/ZnO	1.626	27.53	86.39	28.06	Simulation	^43^
NiO/CH_3_NH_3_SnI_3_/PCBM	0.975	33.45	70.33	22.95	Simulation	^62^
NiO/CH_3_NH_3_PbI_3_/ZnO	1.048	22.17	76.78	17.84	Simulation	^25^
NiO/CH_3_NH_3_PbI_3_/ZnO	0.990	21.00	76.10	15.80	Simulation	^83^
Cu_2_O/ CH_3_NH_3_PbI_3_/PCBM	1.300	22.40	85.50	25.00	Simulation	^84^
ZnO/CH_3_NH_3_SnI_3_/Cu_2_O	1.03	30.14	85.58	26.55	Simulation	^63^
Cu_2_O/CH_3_NH_3_PbI_3_/ZnO	0.700	12.58	0.63	6.02	Experimental	^85^
NiO/CH_3_NH_3_PbI_3_/ZnO	0.680	10.86	0.67	5.02	Experimental	^85^
ZnO/CH_3_NH_3_SnI_3_/Cu_2_O	0.637	46.569	31.17	9.27	Simulation	^57^
Cu_2_O/CH_3_NH_3_SnI_3_/ZnO	1.0867	33.4942	82.88	30.17	Simulation	This work

## Conclusion

The toxic-free CH_3_NH_3_SnI_3_ as light harvesting material is explored in this study. A heterojunction planar perovskite solar cell with an inverted structure Glass/Cu_2_O/CH_3_NH_3_SnI_3_/ZnO/Al was simulated, optimized and analyzed in this paper. In relation to various photovoltaic parameters such as the work function of the back contact electrodes, thickness of the HTM layer, absorber and the ETM layers, and the absorber’s doping concentration were optimized. The thickness of the HTM, absorber layer and ETM were optimized to 40 nm, 1200 nm and 200 nm respectively. The optimized structure produced an enhanced Voc of 1.0867 V, J_SC_ of 33.4942 mA/cm^2^, FF of 82.88% and PCE of 30.17% respectively. The results indicate that an increase in doping concentration of the absorber increased the Voc, FF and PCE but decreased the J_SC_ of the solar cell. The interface between the ETM/back-electrode requires a cheap and low work function metal for enhanced performance. The n-type TCO-free inverted CH_3_NH_3_SnI_3_-based PSC provides a potential path to attaining simple, eco-friendly, cheap and highly efficient perovskite solar cell device using all-inorganic transport materials.

## Data Availability

The data that support the findings can be made available upon reasonable request from the corresponding author on emmanuel.nyiekaa@eng.uniben.edu.
